# Gonococcal Subcutaneous Abscess and Pyomyositis: A Case Report

**DOI:** 10.1155/2012/790478

**Published:** 2012-08-05

**Authors:** Anupop Jitmuang, Adhiratha Boonyasiri, Nukool Keurueangkul, Amornrut Leelaporn, Amorn Leelarasamee

**Affiliations:** ^1^Division of Infectious Disease and Tropical Medicine, Department of Medicine, Faculty of Medicine Siriraj Hospital, Mahidol University, Bangkok 10700, Thailand; ^2^Department of Medicine, Faculty of Medicine Siriraj Hospital, Mahidol University, Bangkok 10700, Thailand; ^3^Department of Microbiology, Faculty of Medicine Siriraj Hospital, Mahidol University, Bangkok 10700, Thailand

## Abstract

Disseminated gonococcal infection (DGI) is an uncommon complication of *Neisseria gonorrhoeae* infection, its manifestation varies from a classic arthritis-dermatitis syndrome to uncommon pyogenic infections of several organs. Herein, we reported atypical presentation of DGI with subcutaneous abscess of right knee, pyomyositis of right lower extremity, and subsequently complicated by *Escherichia coli* pyomyositis. This infection responded to appropriate antimicrobial therapy and prompt surgical management with good clinical outcome.

## 1. Introduction

*Neisseria gonorrhoeae* is a fastidious gram-negative diplococci. It is an important cause of cervicitis, urethritis, and pelvic inflammatory disease (PID) [1]. This organism also causes septic arthritis or a distinct syndrome of disseminated gonococcal infection (DGI), with tenosynovitis, skin lesions, and polyarthralgia [2]. The author reported a patient who had atypical manifestation of DGI complicated by *Escherichia coli* pyomyositis.

## 2. Case Presentation

**Figure 1:**
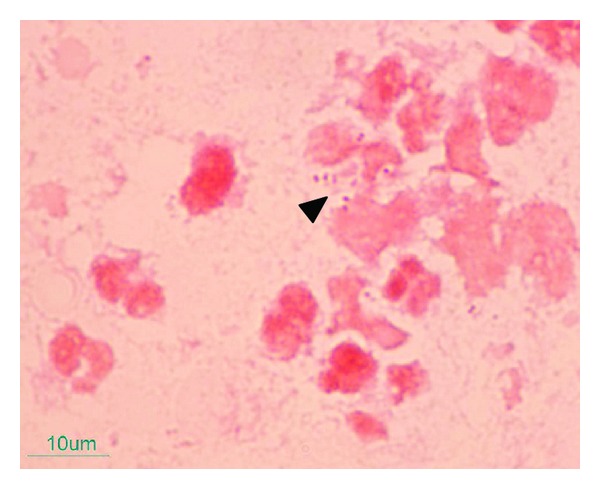
Gram stain of pus specimens obtained from aspiration and incisional drainage of right knee joint showed moderate amounts of gram-negative kidney-shaped diplococci (arrowhead). Pus culture on chocolate agar grew *Neisseria gonorrhoeae*.

**Figure 2:**
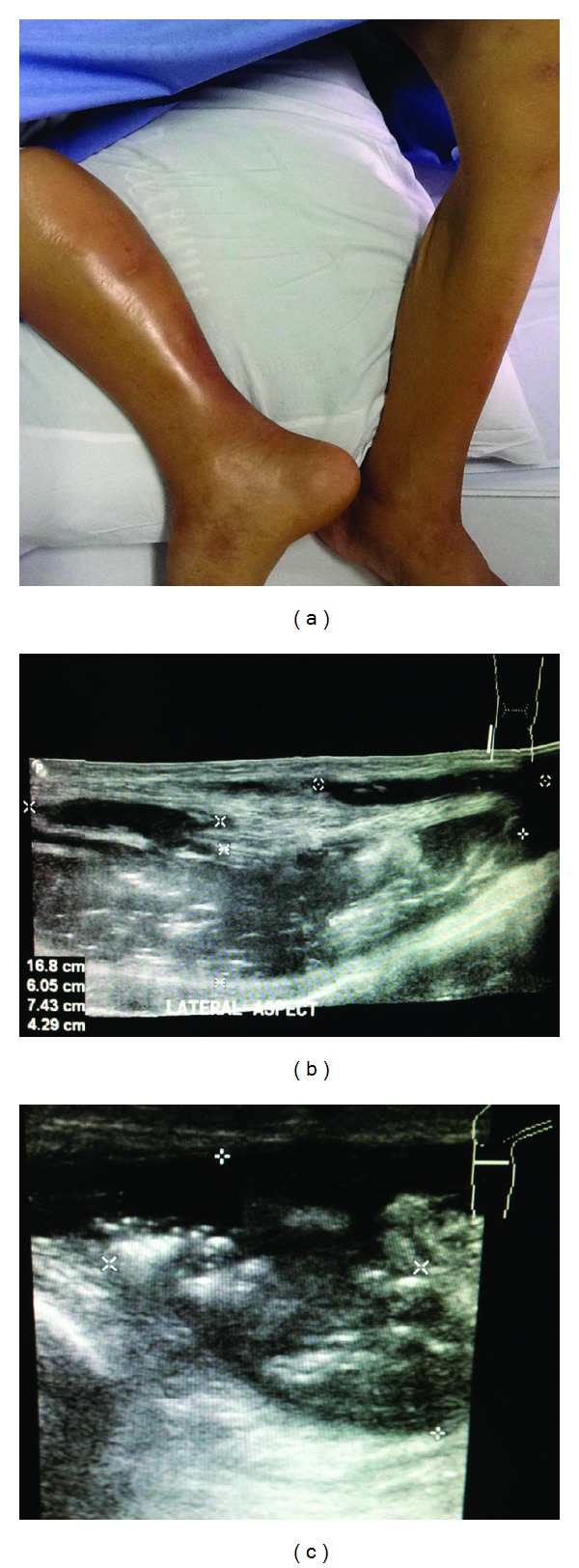
(a) After antimicrobial treatment, there was progressive swelling of right knee extended downward to the calf area, (b, c) ultrasonography of right leg discovered large space of air-filled, heterogeneous multiloculated peri- and intramuscular abscesses, sized 5.4 × 8.6 × 17 centimeters in diameter, extended from popliteal area to ankle level.

A 48-year-old woman with a poorly controlled diabetes mellitus for 12 years, presented with acute severe right knee pain and fever for 7 days. Three weeks before the onset, she fell on the ground accidentally and developed right knee pain. However, she was able to walk after the event and there was no open wound nor knee swelling. A physician provided a short slab for right knee immobilization, but her knee pain was progressive and she was unable to mobilize or leave her bed for one week before admission. She also complained of perianal pain during this illness. At Siriraj Hospital, body temperature was 38.2°C, pulse rate 102/minute, blood pressure and respiratory rate were normal. Her right knee was swollen, fluctuated on the lateral sides with diameter about 7 × 15 centimeters (cm), and marked tenderness and warmth. Anal examination found a draining abscess on the left side of perianal area, sized about 3 × 4 cm. Others were unremarkable. An aspiration of the right knee revealed frank pus. Plain radiography of the right knee was unremarkable. Blood sugar was 413 mg/dL, complete blood count showed hemoglobin of 7.5 g/dL, hematocrit 23.6%, white blood cell count of 23,180 cell/mm^3^ (neutrophil 88.2%, lymphocyte 4.3%, monocyte 4.4%), platelets count of 547,000 cell/mm^3^, ESR 102 mm/hr, and CRP 330 mg/L. Serum BUN and creatinine were within normal limits. She was admitted to the hospital and ceftriaxone 2 g/day with clindamycin 1,800 mg/day were empirically commenced. The surgeon performed incision and drainage (I&D) of the right knee abscess and perianal abscess on the first day of hospitalization. Operative findings showed 300 mL of subcutaneous pus around the right knee without connection to the joint cavity, and 20 mL of pus drained from perianal and intersphincteric abscesses. Gram stain of both specimens of pus showed moderate gram-negative diplococci as shown in Figure 1 and culture on chocolate agar grew *Neisseria gonorrhoeae* with positive beta lactamase testing. The organism was susceptible to ceftriaxone, ciprofloxacin, and tetracycline, but resistant to penicillin by disc diffusion method. Strain and serotype identification of *Neisseria* spp. were not tested in the hospital. Gram stain of pus from perianal abscess showed polymicrobial micro-organisms with gram-negative diplococci, gram-positive cocci in pairs, and rare gram-positive rods. The pus culture grew mixed microorganisms without *N. gonorrhoeae*. All blood cultures were negative. Disseminated gonococcal infection with polymicrobial perianal and intersphincteric abscesses were diagnosed. This patient denied previously multipartners sexual activity, receptive anal intercourse, and prior sexually transmitted diseases. She has been in menopausal period for two years and has been living with her healthy husband. Her last sexual intercourse was 2 months before admission. Atrophic vaginal mucosa and minimal mucus cervical discharge were identified from per vaginal examination and no microorganisms grew from cervical swab cultures. Nucleic acid amplification test (NAAT) of the vaginal discharge was negative for *N. gonorrhoeae* and *Chlamydia trachomatis. *The NAAT of rectal specimens was not approved to detect *N. gonorrhoeae* and *C. trachomatis* coinfection by our regulatory unit. The Anti-HIV antibody test and VDRL were also nonreactive. Serum C3 and C4 complements levels were 70.5 mg/dL (normal range; 87–177) and 23.8 mg/dL (normal range; 7–40), respectively. The initial antibiotics were continued and doxycycline 200 mg/day was added for 7 days for potential chlamydial coinfection. Insulin injection was used for control hyperglycemia. The anal abscesses resolved, however, fever was temporarily subsided. Besides there was persistent pus drainage from the incised wound of right knee and progressive swelling extended downward to calf area (Figure 2(a)). Ultrasonography of the right leg was done and discovered large multiloculated peri- and intramuscular abscesses of the right calf as shown in Figures 2(b) and 2(c). Repeated surgical debridements were performed on day 8 and day 16 of hospitalization, there was foul-smell pus draining from muscles of posterior part of right lower thigh and right calf. Pus culture grew moderate amount of *Escherichia coli*, then intravenous meropenem was then substituted for treatment of complicated pyomyositis. Fever, swollen right leg, and pus drainage were resolved, then the wound was resutured. Meropenem was discontinued after 14 days of therapy. The patient was discharged from the hospital on day 34 of hospitalization. Two weeks later, the patient's condition almost returned to normal and anemia was improving from iron supplementation. Her husband was not available for investigation of gonococcal infection because he had been working in another province.

## 3. Discussion 

The 100% Condom Use Program (CUP) in Thailand since 1990 has reduced the incidence of gonorrhea from 3.2–4.5 per 1,000 population between year 1982–1989 to 0.09–0.1 per 1,000 population between year 2000–2005. Kilmarx et al. and Srifeungfung et al. surveyed prevalence of gonococcal urogenital infection in pregnant women and HIV seropositive patients in Thailand and found the prevalence of 0.2% and 1.3%, respectively [3, 4]. However, there is an increasing trend of sexually transmitted infections among high-risk group such as HIV-infected patients, teenage with multiple sexual partners, and men who have sex with men (MSM). These groups are major reservoirs of transmission. A recent systematic review demonstrated overall point prevalence of gonorrhea of 9.5% among people living with HIV/AIDS, and 13% of the point prevalence from Thai MSM clinic [5]. Thus, gonorrhea should be aware as a re-emerging disease now. 

Disseminated gonococcal infection (DGI) results from bacteremic dissemination of *N. gonorrhoeae*. It was estimated to occur in 0.5% to 3% of patient with gonorrhea [6]. Menstruation and humoral immunity especially terminal complement components deficiency are major risk factors for DGI [2], the reported case showed merely low borderline C3 complement level. Up to 13% of patients with DGI have complement deficiencies [2], while diabetes mellitus as was the case report has no correlation with this condition. Moreover, some serotypes such as the porin 1A serotype was associated with disseminated infection. Porins can downregulate complement system of host cell [2] but serotype assay for *Neisseria* spp. is not available in our institute. Septic arthritis and a syndrome of polyarthritis and dermatitis are the predominant features. However, meningitis, osteomyelitis, septic shock, and acute respiratory distress syndrome which were rare conditions could be presented. The reported patient initially presented with fever and subcutaneous abscess of right knee including perianal and intersphincteric abscesses that were unusual manifestations. Gonococcal skin and soft tissue infections have been reported by several studies since 1926 [7] and also from our institute [8, 9] but might be forgotten due to rare presentation of the case after 100% condom campaign. Clinical presentation varied from subcutaneous abscess to pyomyositis with or without genitourinary symptoms. An old-time review from Newburger B reported several case series of gonococcal intramuscular abscess and most patients had preceding genitourinary infection. The author also postulated 3 mechanisms of intramuscular infection, that is, primary intramuscular infection, lymphatic drainage from arthritic focus, and secondary infection due to endocarditis or pyemia [7]. Additionally, the same author described cases of gonococcal subcutaneous abscesses that were probably extended from adjacent arthritic sites [7]. The presented case developed subcutaneous abscess on right knee without preexisting symptoms of cervicitis or neighboring arthritis. Hence, we believed it was secondary to disseminated infection. Identifying gram-negative diplococci in the pus obtained from perianal abscess and intersphincteric abscess indirectly showed that these sites were likely to be the primary focus albeit the pus cultures yielded negative for *N. gonorrhoeae*. Identification of gonococci could be veiled by overgrowth of gastrointestinal bacterial flora. Patients with DGI usually have negative urogenital symptoms and about 50% of the cases had positive blood culture, especially in arthritis-dermatitis syndrome [2, 10]. Although the presented case was given an appropriate antimicrobial therapy, the infection progressed to extensive pyomyositis in lower part of right thigh and right calf. These complications were possibly caused by delayed prompt therapy with antibiotics and surgical drainage. Gonococcal pyomyositis had been reported in a few literatures, all three patients were young and presented with subacute progressive pain ranged between 2-3 weeks [11–13] as showed in this case report. The involved muscles were left calf, right biceps, and right obturator internus, consecutively. Surgical drainage identified purulent discharge with positive culture for gonococci in all cases [11–13]. One patient had no prodomal urogenital symptoms like this reported case. Undoubtedly, *N. gonorrhoeae* isolates were not able to identify from muscular abscesses owing to ongoing ceftriaxone treatment and overgrowth of *E. coli*. Poorly controlled hyperglycemia may be a contributing factor for the *E. coli* pyomyositis in the patient. 

*N. gonorrhoeae* strain of this case showed susceptible to standard antibiotics except for penicillin. In Thailand, surveillance of antibiotic resistance in *N. gonorrhoeae* showed quinolone-resistance *N. gonorrhoeae* (QRNG) or reduced susceptibility in excess of 90% [14]. Though susceptibility to ceftriaxone was not abruptly changed, treatment failure occurred while receiving oral third-generation cephalosporins. Hence,* in vitro *criteria used to categorize the clinical importance of gonococci with different ceftriaxone and oral cephalosporin MIC levels should be revised [14]. Because of breakthrough *E. coli* pyomyositis, ceftriaxone was changed to meropenem. Meropenem itself is very active against both positive and negative beta-lactamase producing *N. gonorrhoeae* [15, 16] and our patient had good clinical outcome. Anyhow, we could not provide her partner with a gonococcal screening for prevention of further transmission.

## 4. Conclusion 

Gonorrhea outside urogenital tract should be aware as a reemerging disease and can be presented with dermatitis-arthritis syndrome or pyogenic polyarthritis or subcutaneous abscess. Whenever a patient who has a sexually active behavior presented with pyogenic skin and soft tissue infections with or without prodomal urogenital symptoms, DGI should be considered in the differential diagnosis. Early diagnosis and management of this condition produce a good outcome.
